# Reduced Resting-State Functional Connectivity of the Somatosensory Cortex Predicts Psychopathological Symptoms in Women with Bulimia Nervosa

**DOI:** 10.3389/fnbeh.2014.00270

**Published:** 2014-08-04

**Authors:** Luca Lavagnino, Federico Amianto, Federico D’Agata, Zirui Huang, Paolo Mortara, Giovanni Abbate-Daga, Enrica Marzola, Angela Spalatro, Secondo Fassino, Georg Northoff

**Affiliations:** ^1^Department of Neuroscience, AOU San Giovanni Battista, Turin, Italy; ^2^Institute of Mental Health Research, University of Ottawa, Royal Ottawa Healthcare Group, Ottawa, ON, Canada; ^3^Graduate Institute of Humanities in Medicine, Taipei Medical University, Taipei, Taiwan; ^4^Brain and Consciousness Research Center, Taipei Medical University-Shuang Ho Hospital, New Taipei City, Taiwan; ^5^Research Center for Mind, Brain and Learning, National Chengchi University, Taipei, Taiwan; ^6^Department of Psychology, National Chengchi University, Taipei, Taiwan; ^7^Centre for Cognition and Brain Disorders (CCBD), Normal University Hangzhou, Hangzhou, China

**Keywords:** bulimia, eating disorders, fMRI, resting state, functional connectivity, somatosensory network

## Abstract

**Background:** Alterations in the resting-state functional connectivity (rs-FC) of several brain networks have been demonstrated in eating disorders. However, very few studies are currently available on brain network dysfunctions in bulimia nervosa (BN). The somatosensory network is central in processing body-related stimuli and it may be altered in BN. The present study therefore aimed to investigate rs-FC in the somatosensory network in bulimic women.

**Methods:** Sixteen medication-free women with BN (age = 23 ± 5 years) and 18 matched controls (age = 23 ± 3 years) underwent a functional magnetic resonance resting-state scan and assessment of eating disorder symptoms. Within-network and seed-based functional connectivity analyses were conducted to assess rs-FC within the somatosensory network and to other areas of the brain.

**Results:** Bulimia nervosa patients showed a decreased rs-FC both within the somatosensory network (*t* = 9.0, df = 1, *P* = 0.005) and with posterior cingulate cortex and two visual areas (the right middle occipital gyrus and the right cuneus) (*P* = 0.05 corrected for multiple comparison). The rs-FC of the left paracentral lobule with the right middle occipital gyrus correlated with psychopathology measures like bulimia (*r* = −0.4; *P* = 0.02) and interoceptive awareness (*r* = −0.4; *P* = 0.01). Analyses were conducted using age, BMI (body mass index), and depressive symptoms as covariates.

**Conclusion:** Our findings show a specific alteration of the rs-FC of the somatosensory cortex in BN patients, which correlates with eating disorder symptoms. The region in the right middle occipital gyrus is implicated in body processing and is known as extrastriate body area (EBA). The connectivity between the somatosensory cortex and the EBA might be related to dysfunctions in body image processing. The results should be considered preliminary due to the small sample size.

## Introduction

Bulimia nervosa (BN) is a prevalent and difficult to treat eating disorder (Fassino and Abbate-Daga, 2013). It shares with anorexia (AN) an overvaluation of the perception of shape and weight of the body, such that patients’ self-worth depends largely or exclusively on it (Fairburn and Harrison, 2013). Both psychodynamic and cognitive–behavioral theorists concur that this aspect is a central feature of eating disorders (Skårderud, 2007; Abbate-Daga et al., 2010; Spangler and Allen, 2012).

Different components of body image disturbance have been distinguished, like the altered estimation of one’s own body size as opposed to the attitude toward one’s own body in terms of an emotional evaluation (Slade, 1994; Skrzypek et al., 2001). This evaluation is often characterized by deep dissatisfaction in patients with eating disorders. Body image disturbances have been shown to precede the onset of eating disorders (Killen et al., 1996) and to predict maintenance (Bohon et al., 2009) and relapse (Keel et al., 2005). More specifically, body weight and shape dissatisfaction have been shown to be stronger in BN patients compared to other eating-disordered patients (Abbate-Daga et al., 2010). These symptoms have been suggested to be linked to alterations in the lateral occipital cortex in bulimia (Mohr et al., 2011), but their neurobiological correlates are still incompletely understood. Interoceptive awareness, defined as a person’s lack of confidence in recognizing and identifying emotions and sensations of hunger and satiety, has also been traditionally considered a relevant dimension in the psychopathology of eating disorders (Garner et al., 1983). Interoceptive deficits have been shown to be present in bulimic subjects in a recent study, and they have been hypothesized to play a role in the development and maintenance of bulimic symptoms (Klabunde et al., 2013). However, the exact neuronal mechanisms underlying the key abnormalities of BN in body perception and bulimic symptoms as they are assessed with standard inventories like the Eating Disorder Inventory (Garner et al., 1983) (e.g., the dimensions of bulimia, body dissatisfaction, and interoceptive awareness) remain unclear.

The neurobiological underpinnings of eating disorders have been the subject of an increasing number of studies (van den Eynde and Treasure, 2009; Eddy and Rauch, 2011).

In AN, several studies point to abnormalities in the insular cortex, cingulate cortex, and basal ganglia (Frank, 2012); recent work indicates alterations in the salience network, in the default mode network, and in the sensorimotor and visuospatial networks (Favaro et al., 2012; McFadden et al., 2014).

The insular cortex has also been shown to be altered in bulimia (Frank et al., 2013), in addition to structural abnormalities in the orbitofrontal cortex and the dorsal striatum (Schäfer et al., 2010; Frank et al., 2013).

It is likely that dysfunction of cognitive and emotive process depends on distributed regions, spanning multiple lobes and their respective neural networks (Menon, 2011). This requires an investigation of neural networks and their functional connectivity, which can be reliably studied with resting-state paradigms (Seeley et al., 2007).

In particular, there are reasons to believe that the executive (Uher et al., 2004), default mode (Cowdrey et al., 2014), salience (Lee et al., 2014), and somatosensory (Favaro et al., 2012) networks might play a role in the psychopathology of eating disorders.

Two recent studies of resting state in BN demonstrated changes in the cerebellum (Amianto et al., 2013b) and in the salience network (Lee et al., 2014). This leaves open, however, the role of resting-state functional connectivity (rs-FC) in the somatosensory network in BN and its relationship to the disturbances in body perception.

The aim of the present study consisted first in investigating the rs-FC in the somatosensory network and in other networks that might be altered in eating disorders, and secondly in testing how that is related to three psychopathological symptoms of BN: bulimia (a defining feature of the disorder), body dissatisfaction, and interoceptive awareness (two body-related symptom dimensions).

If alterations of body image are central in the psychopathology of eating disorders as the theories that we previously mentioned suggest, it should be expected that alterations in the somatosensory network should be found and should also negatively affect the function of other brain areas. Therefore, we hypothesized that alterations should be present in the rs-FC of the somatosensory network and of the other networks, but also that eating disorder symptom scales should specifically correlate with the within-network functional connectivity of the somatosensory cortex and with its rs-FC with other brain regions.

A two-step approach was used: first, the average within-network connectivity of each network was studied; then a seed-based analysis was used to compute the rs-FC of one seed region for each of the networks that were found to be altered in the first analysis. A previous study in part of the present sample of bulimic patients identified a region in the somatosensory network (the left paracentral lobule) that showed higher gray matter density compared to controls (Amianto et al., 2013a). We hypothesized that the anatomical alteration that we found in the previous study might be linked with anomalies in the rs-FC with other brain areas. Therefore, we used this region as the seed region for the seed-based analysis in the somatosensory network.

## Materials and Methods

Sixteen women who were assessed after their first contact at the Center for Eating Disorders of the University of Turin and diagnosed with BN and 18 matched healthy women (contacted through local advertisement) were recruited for the study. A formal diagnosis of BN was established through a clinical interview by a psychiatrist expert in the assessment and treatment of eating disorders following DSM-IV-TR criteria (American Psychiatric Association, 2000). All subjects were right-handed. Exclusion criteria for both patients and controls were major medical illness, neurological disorders, head trauma, use of psychotropic medications, or psychotherapeutic treatments in the last 6 months, presence of other relevant psychiatric diagnoses except mild depressive symptoms (e.g., not meeting criteria for an axis I mood disorder diagnosis), or any contraindication for MRI. Both groups of participants completed a self-report questionnaire investigating eating disorder symptoms (Eating Disorder Inventory, EDI-II, Garner et al., 1983).

All patients and controls gave their written informed consent to the study. The study was approved by the Ethical Committee of the San Giovanni Battista Hospital, Turin.

### fMRI data acquisition

Data were collected at the Neuroscience Department AOU San Giovanni Battista, Turin, Italy on a Philips Achieva 1.5 T scanner (Erlangen, Holland) equipped with dual gradient system (Quasar, Philips) of 40 mT/m and a Sense high-field, high-resolution eight-channel head coil optimized for functional imaging. The resting-state scan comprised 200 continuous functional volumes (repetition time = 1966 ms, echo time = 50 ms, flip angle = 90°, 24 axial slices, matrix = 128 × 128, slice thickness = 5 mm, acquisition voxel size = 1.8 mm × 1.8 mm × 6 mm, field of view = 25 cm). The total acquisition time was 6 min and 33 s; the patients were scanned with their eyes closed. Subjects were instructed to report anxiety feelings during the scanning on a scale ranging from no anxiety, to minimal tolerable anxiety, mildly disturbing, disturbing, severe anxiety, panic attack. All subjects reported an experience of anxiety feelings ranging from minimal tolerable anxiety (*n* = 2 patients, three controls) to no anxiety at all (the rest of the sample), so anxiety was not accounted for the elaboration of the data. All participants reported keeping their eyes closed and being awake during the scanning.

### Data processing

The pre-processing steps were implemented in AFNI (Cox, 1996) (http://afni.nimh.nih.gov/afni). Alignment (head motion correction), slice timing correction, temporal standardization, and spatial smoothing (6 mm) were performed on the functional images. To denoise our data, we regressed out the signal from the white matter and cerebrospinal fluid to minimize the physiological aliasing, such as respiration in the low frequency bands in the rs-FC analyses (Fox and Raichle, 2007). In addition, we also removed out the motion artifacts by using the estimated six-dimensional time series of head motion (by AFNI program 3dvolreg) as covariates during the FC analysis. The estimation of head motion was tested with a procedure previously described by Zang et al. (2007). The magnitude of head motion for six parameters (three for shift and three for rotation) was obtained for each subject and the averaged head motion parameters were calculated. A *t*-test for independent samples was obtained in order to test for differences between the groups. The result of the test was non-significant both for rotation (*t* = 0.2; *p* = 0.8; average movement × TR = 0.05º) and for shift (*t* = 0.6; *p* = 0.5; average movement × TR = 0.1 mm), suggesting that no significant differences in motion could be demonstrated between the groups. Zang and colleagues suggest to exclude from the analyses subjects with head motion exceeding 4 SDs (in our sample: rotation >0.08°; shift >0.16 mm). None of our subjects exceeded this threshold; therefore, all subjects were included in the analyses. The images were transformed into Talairach space (Talairach and Tournoux, 1988) and they were resampled to 3 mm × 3 mm × 3 mm. The data were then filtered with a band-pass filter maintaining signals between 0.01 and 0.1 Hz, which is thought to reflect neuronal fluctuations (Biswal et al., 1995; Fox and Raichle, 2007; Zhang and Raichle, 2010).

The rs-FC was computed between the averaged time series of a given seed region and all voxels in the brain for the selected frequency range (0.01–0.1 Hz). The estimated head motion and the mean time series from the white matter and the cerebrospinal fluid were used as covariates in the procedure to avoid artifacts (Fox et al., 2005; Saad et al., 2012). The rs-FC maps from individual subjects were then transformed to *Z* values with Fisher’s transformation for group-level analyses. All group-level analyses were performed with age, BMI (body mass index), and depressive symptoms as nuisance variables.

### Within-network connectivity

In order to compute the within-network connectivity, the first step was to identify the key nodes of the selected networks, to be used as seed regions in the subsequent analysis. We selected one seed region for each network of interest: the posterior cingulate cortex (PCC) for the default mode network (Greicius et al., 2003), the dorsal anterior cingulate gyrus for the salience network (Seeley et al., 2007), the left dorsolateral prefrontal cortex for the executive network (Curtis and D’Esposito, 2003), and the left postcentral gyrus for the somatosensory network (Biswal et al., 1995). The seed regions were defined by means of the AFNI atlas (Eickhoff et al., 2005). We derived functional maps of connectivity of regions significantly co-activated with the seeds in the whole sample, and six ROIs were obtained from the rs-FC map of each seed region to characterize the relative network. Coordinates for the seeds and the ROIs are provided in Table S1 in Supplementary Material; captions of the resulting networks are provided in Figures S1–S4 in Supplementary Material.

We then proceeded to extract time series from all the ROIs and calculated the rs-FC between all the nodes of a given network. The average connectivity of a network was computed averaging the rs-FC between all the pairs of nodes within a network (i.e., 15 pairs for 6 regions of each network). An ANOVA for independent samples was then calculated to establish differences in the within-network average connectivity between the groups. The ANOVA was corrected for multiple comparisons to a significant level of *p* < 0.05 (Bonferroni correction for four comparisons between patients and controls in the four networks). In networks where such differences were found, correlations between the eating disorder psychopathology scales and the average within-network connectivity were tested.

### Seed-based study: The FC of the paracentral lobule

A previous Voxel-Based Morphometry study that was based on part of the present patient sample (Amianto et al., 2013a) identified a region in the left paracentral lobule as characterized by higher gray matter density in bulimic patients compared to controls. This region appears to be within the somatosensory network as defined by the rs-FC of the postcentral gyrus in our sample, previously calculated (see Figure S5 in Supplementary Material for a visualization of the region inside the network). This region was chosen as the seed region for a subsequent whole-brain analysis, testing for differences in the rs-FC of the paracentral lobule with the rest of the brain. The same procedures described above for obtaining rs-FC were applied. A *t*-test was computed to reveal differences in rs-FC between patients and controls. A dual thresholding of both type I error and cluster size was performed to correct for multiple comparison. The *t*-maps were thresholded at a corrected *p* value <0.05 at the cluster level obtained from the procedure (minimal cluster size 86 voxels). The cluster size was obtained with a Monte Carlo simulation as implemented in AlphaSim, an application of AFNI. The smoothness used in AlphaSim was the average smoothness of the EPI time series across subjects. A level of 5000 permutations was specified.

The averaged values of the rs-FC in the resulting regions were tested for correlation with three psychometric scales of the Eating Disorder Inventory (bulimia, body dissatisfaction, and interoceptive awareness) to investigate the presence of relationships between the rs-FC of the paracentral lobule and eating disorder psychopathology. Age, education, and depressive symptoms were used as covariates in the correlation to control for confounding. To avoid Type I error, the correlations were further tested with bootstrapping by creating 500 bootstrap samples, and confidence intervals were computed. All statistical analyses were carried out with PASW statistics 20.0 (SPSS Inc.).

## Results

### Sample

**Figure 1:**
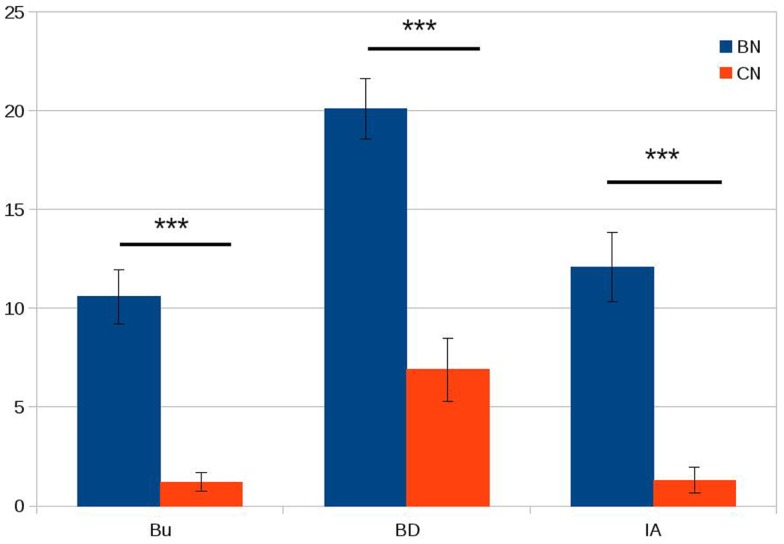
**Psychopathology scores in patients with bulimia (BN) and control subjects (CN)**. Bu = bulimia; BD = body dissatisfaction; IA = interoceptive awareness. On the *Y* axis, we plotted the average scores for the bulimia and control groups. Error bars represent die standard error. ***P < 0.001.

We did not observe any significant differences between bulimic subjects and healthy controls in age, BMI, or education (see Table 1). The three eating psychopathology scales (bulimia, body dissatisfaction, and interoceptive awareness) showed statistically significant differences between the two groups as expected with bulimic subjects scoring significantly higher than healthy subjects (see Table 1; Figure 1 for details). Eight of the 16 bulimic patients showed depressive symptoms that did not meet criteria for an axis I diagnosis; none of the control subjects showed depressive symptoms.

**Table 1:** **Demographic and psychopathology variables**.

	CN (*n* = 18)		BN (*n* = 16)		*T*	*P* value
	Mean	SD	Mean	SD	
Age (years)	23	3	23	5	−0.4	0.96
Education (years)	16	2	15	2	−1.2	0.22
BMI	21	2	22	2	0.45	0.65
EDI II Bulimia	1	2	11	5	18.5	<0.001
EDI II Body Dissatisfaction	7	7	20	6	32	<0.001
EDI II interoceptive awareness	1	3	12	7	19	<0.001
Length of illness (months)	–	–	17	12		–

*CN, healthy control women; BN, bulimic women; BMI, body mass index; EDI II, Eating Disorder Inventory; T, t test*.

### Within-network connectivity

**Figure 2:**
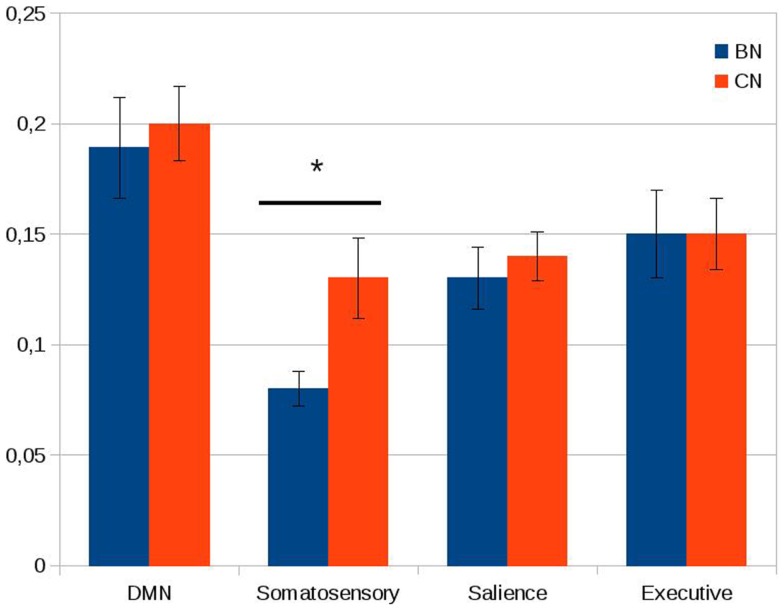
**Within-network connectivity of the default mode network (DMN), somatosensory network, salience network, and executive network**. On the *Y* axis, we plotted the average of the Pearson correlations between the time series of all the couples of nodes inside each network. Error bars represent the standard error. Note the significant difference between the two groups in the somatosensory network. *P < 0.05 (Bonferroni correction applied).

The within-network rs-FC for each network was computed averaging the rs-FC of all the pairs of key regions inside of a network. A significant decrease was found in BN patients compared to controls in the within-network connectivity of the somatosensory network (*t* = 9.0, df = 1, *P* < 0.05 with Bonferroni correction for multiple comparison). In contrast to the somatosensory network, we did not observe any significant between-group differences in the average within-network connectivity of the default mode network, the executive network, and the salience network (see Figure 2).

### Seed-based study of the paracentral lobule

**Figure 3:**
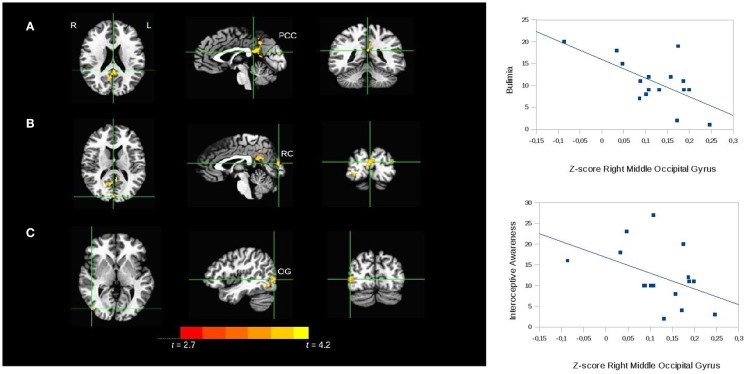
**Seed-based analysis of the functional connectivity of the paracentral lobule, comparing patients with bulimia nervosa with healthy controls**. Areas of significant difference (cluster-wise *p* < 0.05 corrected; threshold at *t* > 2.7) between bulimic patients and controls: **(A)** left posterior cingulate cortex (PCC) (peak = 1, 40, 20; *t* = 3.7; Brodmann area 29); **(B)** right cuneus (RC peak = −1, 85, 14; *t* = 4.2; Brodmann area 18). **(C)** right middle occipital gyrus (OG) (peak = −43, 76, 2; *t* = 3.6; Brodmann area 19); analyses conducted with age, BMI, and depressive symptoms as covariates. L, left; R, right. The graphs show the relationship between the *Z*-scores of the functional connectivity in the area **(C)** and the psychopathology scales. The correlations were confirmed with bootstrapping.

The seed-based analysis revealed a decrease in the rs-FC of the paracentral lobule in BN subjects compared to controls in three regions (*p* = 0.05): the left PCC (peak = 1, 40, 20; *t* = 3.7; Brodmann area 29), the right middle occipital gyrus (peak = −43, 76, 2; *t* = 3.6; Brodmann area 19), and the right cuneus (peak = −1, 85, 14; *t* = 4.2; Brodmann area 18) (see Figure 3 for details).

### Relationship to symptoms

No correlations were found between the within-network connectivity of the somatosensory network in both groups and the psychopathology scales considered.

In the bulimic patients the rs-FC of the right middle occipital gyrus correlated inversely with the bulimia (*r* = −0.4; *P* = 0.02) and interoceptive awareness (*r* = −0.4; *P* = 0.01) subscales of the EDI-II (scatterplots in Figure 3). The bootstrap procedure described in the Section “Materials and Methods” confirmed the significant correlations. No correlations were found with the rs-FC of the right cuneus.

Finally, no correlations were observed between the psychopathology scales and the rs-FC in these regions in control subjects.

## Discussion

In the present study, the within-network connectivity of the somatosensory network was significantly reduced in BN compared to controls. In contrast, our study did not show significant differences in the within-network rs-FC of the other networks (executive, salience, DMN) in BN patients. It is important to emphasize that our approach was to investigate within-network connectivity in the first step, and whole-brain network connectivity only in networks where significant alterations of within-network rs-FC could be found, in order to minimize false-positive findings. Therefore, we cannot exclude that whole-brain connectivity alterations might exist in these networks in BN patients, as a recent study on the rs-FC of the salience network seems to suggest (Lee et al., 2014).

The whole-brain rs-FC of the paracentral lobule is significantly lower in bulimic patients compared to controls in the PCC, in the right middle occipital gyrus, and in the right cuneus. The young age and relatively short duration of illness suggest that these alterations should not be related to effects of a continued state of illness.

Posterior cortical midline structures like the PCC are thought to be involved, among other aspects, in integrating self-referential stimuli in the context of one’s own person (Northoff and Bermpohl, 2004). The somatosensory network is involved in the processing of the stimuli from the own body. Taken together, this seems to suggest that decreased rs-FC between somatosensory network (e.g., paracentral lobule) and PCC may signal decreased self-related processing of the own body. The body and its various somatosensory stimuli might no longer be related to the own self, e.g., the mental representation of one’s self. This interpretation would be consistent with recent work in psychopathology, which proposes that the disturbance in the way in which the body is experienced and integrated in personal identity is central in eating disorders and produces anomalies of eating behavior as a consequence (Stanghellini et al., 2012).

The area of the occipital cortex in which we have found a decrease of rs-FC with the paracentral lobule has been shown to respond selectively to images of human bodies, and has been labeled the extrastriate body area (EBA) (Downing et al., 2001). Our results could indicate a reduced function of this area. This finding would appear consistent with those of Uher and colleagues, who found a decreased activation of the occipitotemporal cortex including the EBA in AN and BN patients during presentations of normal and altered (underweight, overweight) human bodies (Uher et al., 2005). The authors interpreted these results as evidence for a dysfunction in body-image-processing brain circuits in AN and BN patients.

Work in other fields seems compatible with this hypothesis.

In a study by Cazzato et al. investigating the neural underpinnings of the esthetic appreciation of human bodies in healthy volunteers, the authors found a stronger right hemisphere lateralization in women. The authors propose that the right EBA might be involved in body image disorders in eating disorder patients, which is consistent with our results (Cazzato et al., 2014). Moro and colleagues studied patients with lesions involving the EBA and found a specific association with an impaired recognition of the body (Moro et al., 2008).

The finding of a decreased functional coupling between the paracentral lobule and the EBA could also reflect a deficit of multisensory integration between visual and somatosensory information.

We did not find correlations between the psychopathology scales and within-network rs-FC in the somatosensory network, contrary to what we initially hypothesized. The presence of the correlations between eating disorder psychopathology scales and the seed-based rs-FC seems to suggest that the studied symptoms are related to dysfunctions in processes relying on distributed brain regions spanning multiple lobes (Menon, 2011). However, given the small sample size it is important to bear in mind that our results are preliminary and should be confirmed by other studies.

In BN, deficits in interoception have been shown to be stronger than in AN in a study based on the EDI-II (Fassino et al., 2004). Furthermore, decreased interoceptive sensitivity has been recently directly shown in women recovered from BN in a study that used the Heart Beat Perception Task (Klabunde et al., 2013). The authors of the latter study hypothesized that these impairments could lead to binging symptoms. In our sample of bulimic subjects the functional coupling of the paracentral lobule with the EBA correlates inversely with the interoceptive awareness scale, a measure of a person’s lack of confidence in recognizing bodily sensations (Garner et al., 1983), suggesting that interoceptive awareness increases as integration with the visual cortex decreases. Case et al. (2012) proposed that in anorexia there might be a reduced reliance on visual inputs and increased reliance on proprioceptive information, due to dysfunctional multisensory integration. This mechanism might be hypothesized in our sample of bulimic patients as well.

The rs-FC of the paracentral lobule with the EBA was also inversely correlated with the bulimia scale, confirming that the lack of integration between the somatosensory network and the visual cortex could also be related to other symptom domains (Spangler and Allen, 2012).

These findings provide some indications for the development of new treatment approaches to BN.

The deficit in multisensory integration between the somatosensory and the visual networks emphasize the importance of body image in bulimia, and might account for the centrality of body image disturbance in resistance to treatment (Stice, 2002). Treatment strategies that include specific components addressing body image disturbance have been proven to be effective (Trentowska et al., 2013a,b); future studies assessing how these treatments affect the resting-state connectivity in brain regions that process body perception and how the changes might mediate treatment outcomes would greatly advance our knowledge on the disorder.

Another possible future development in the treatment of BN lies in targeting the EBA with neuromodulation techniques, like transcranial magnetic stimulation (TMS).

Studies in bulimia have shown that subcortical pathways are probably impaired in this patient population (Wagner et al., 2010; Frank et al., 2013). These alterations are thought to be related to reward and the ability to identify the emotional implications of stimuli. Deficits in self-regulation and altered perception of body size are on the other hand thought to be due to other abnormalities in cortical structures, notably the inferior frontal regions, the insula, and the lateral occipital cortex (Mohr et al., 2011; Frank et al., 2013; Marsh et al., 2013). Our study adds to this last type of findings. A framework of cortical and subcortical patterns of alterations is gradually emerging as an organizing principle of new findings in the eating disorders neuroimaging literature, and will probably be further developed by future studies. Several limitations should be considered in interpreting our data. The generalizability of our results is limited by the small sample size; for this reason, they should be considered as preliminary findings that need confirmation. The subjects enrolled in the study were not administered as a structured interview to determine the presence of other diagnoses. However, during the initial clinical assessment an initial evaluation was made by a clinical psychiatrist to investigate the presence of other DSM diagnoses and all major domains of psychopathology were systematically investigated using a predefined format. Another limitation of the study is the lack of a more comprehensive assessment of eating disorder symptoms and particularly body image. It might have been relevant to include in the analyses minimum and maximum body weights, since it has been shown that past weight changes influence current body image (Stice, 2002), but this information was not systematically assessed and reported.

Finally, given the small sample size of the study the findings reported should be considered preliminary.

In conclusion, in the present study we found preliminary evidence for a reduction in the functional connectivity of the somatosensory network in BN subjects, both within the network and in other regions of the brain relevant to body image. These alterations appear to be related to the symptoms of BN, and should be addressed in the treatment of BN patients.

## Author Contributions

Luca Lavagnino analyzed the data and wrote the paper, under the supervision and with the participation of Georg Northoff. Federico Amianto wrote the initial research project and co-wrote the paper. Federico D’Agata, Zirui Huang, and Paolo Mortara contributed to data analysis and interpretation; Giovanni Abbate-Daga, Enrica Marzola, Angela Spalatro, and Secondo Fassino contributed to the acquisition of data and writing the discussion. All the authors had access to study data, are responsible for all contents of the article, had authority over manuscript preparation and the decision to submit the manuscript for publication, and approved the submission to the journal.

## Conflict of Interest Statement

The authors declare that the research was conducted in the absence of any commercial or financial relationships that could be construed as a potential conflict of interest.

## Supplementary Material

The Supplementary Material for this article can be found online at http://www.frontiersin.org/Journal/10.3389/fnbeh.2014.00270/abstract
