# Supplementary material for: Reduced Resting-State Functional Connectivity of the Somatosensory Cortex Predicts Psychopathological Symptoms in Women with Bulimia Nervosa
Source: Front Behav Neurosci. 2014 Aug 4;8:270. doi: 10.3389/fnbeh.2014.00270 (PMC4120855; doi:10.3389/fnbeh.2014.00270)
Supplement: Supplementary file 1 [file Data_Sheet1.PDF]

Supplementary Tables

Supplementary Table 1 Key regions of the networks of interest. All locations are expressed in Talairach coordinates.

Default Mode Network

Threshold=94° percentile; corrected p value < 0.01; cluster=80 voxel

**Regions**	**x**	**y**	**z**
left posterior cingulate	4	49	20
right medial frontal gyrus	-4	-43	38
left middle temporal gyrus	43	58	23
right superior temporal gyrus	-43	-16	-15
right middle temporal gyrus	-49	61	26
left middle temporal gyrus	55	10	-3

Executive Network

Threshold=95° percentile; corrected p value < 0.01; cluster=80 voxel

**Regions**	**x**	**y**	**z**
left middle frontal gyrus	40	-25	26
left inferior parietal lobule	52	31	35
right middle frontal gyrus	-43	-37	23
right cungulate gyrus	-7	-10	41
left inferior temporal gyrus	52	52	-3
right inferior frontal gyrus	-46	-7	32

Salience network

Threshold=97° percentile; corrected p value < 0.01; cluster=80 voxel

**Regions**	**x**	**y**	**z**
left cingulate gyrus	1	-16	26
left superior frontal gyrus	28	-43	26
left insula	34	-10	5
right middle frontal gyrus	-25	-40	35
right precentral gyrus	-49	-7	11
right inferior parietal lobule	-61	34	26

Somatosensory Network

Threshold=98° percentile; corrected p value < 0.01; cluster=30 voxel

**Regions**	**x**	**y**	**z**
left postcentral gyrus	37	28	44
right paracentral lobule	-1	13	47
right precentral gyrus	-46	7	29
right pecuneus	-25	52	38
left culmen	22	55	-15
right culmen	-28	40	-18

Supplementary Figures

Key nodes of the studied networks; the procedure for their definition is described in the paper.

Supplementary Figure 1 Default Mode Network

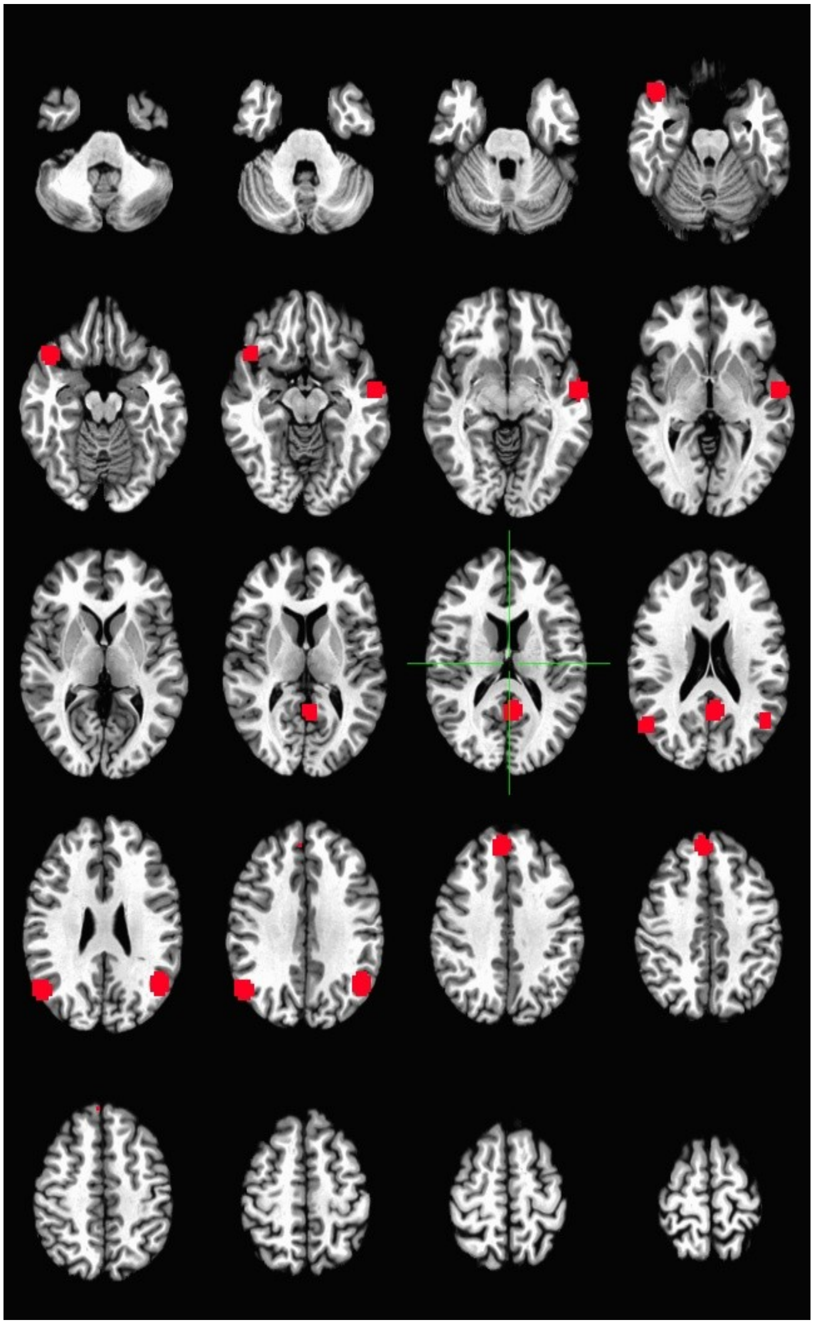

Supplementary Figure 2 Executive Network

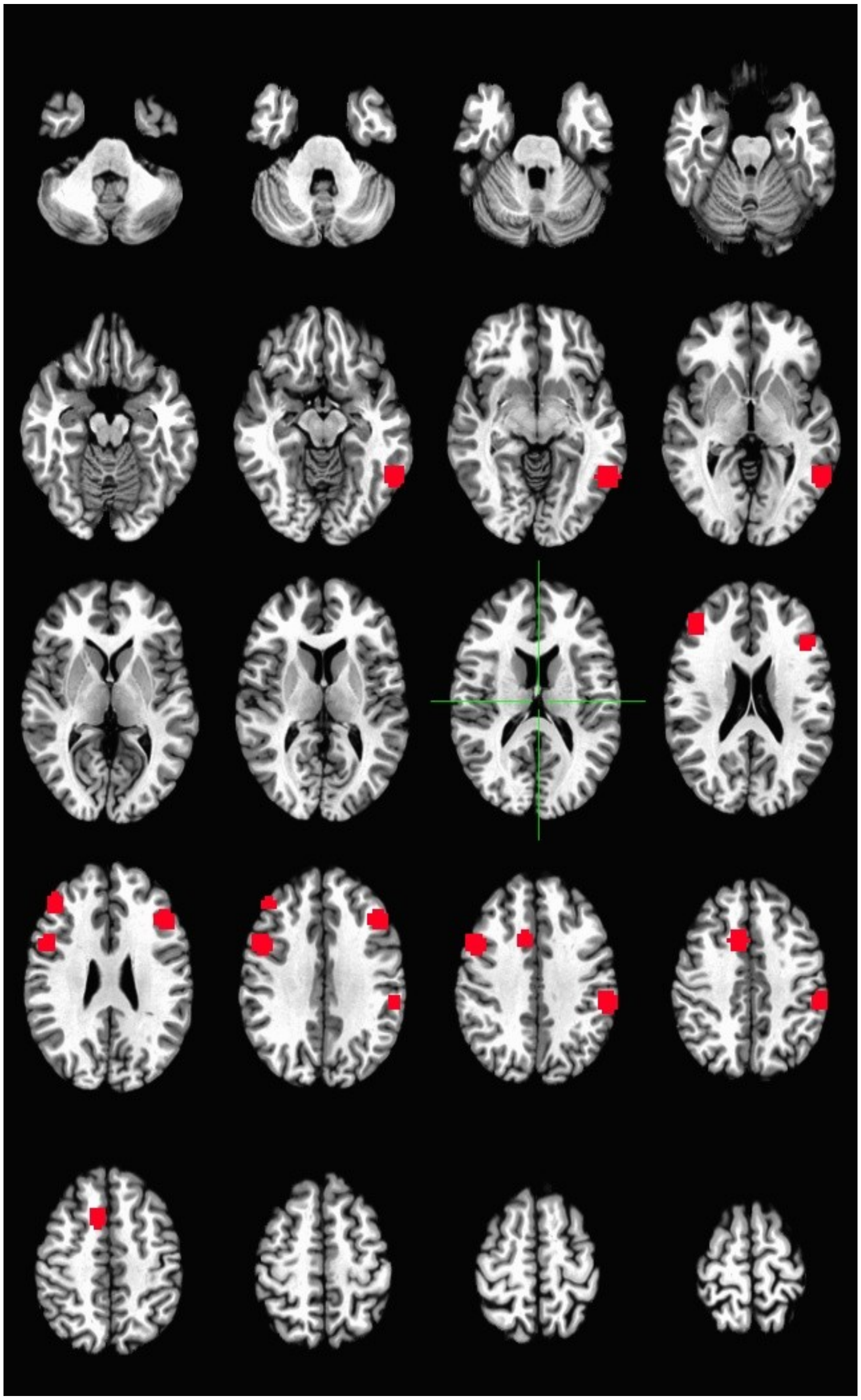

Supplementary Figure 3 Salience Network

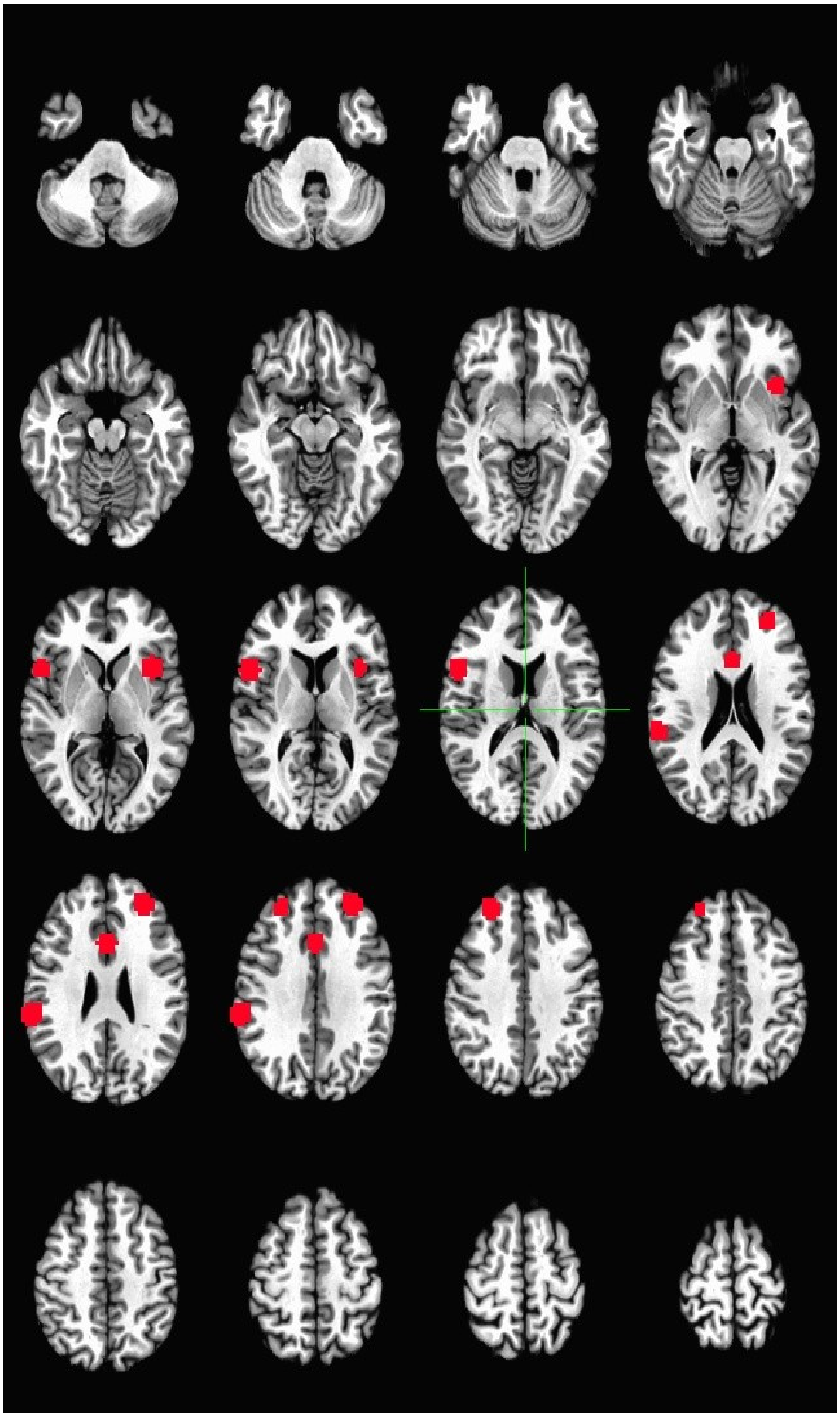

Supplementary Figure 4 Somatosensory Network

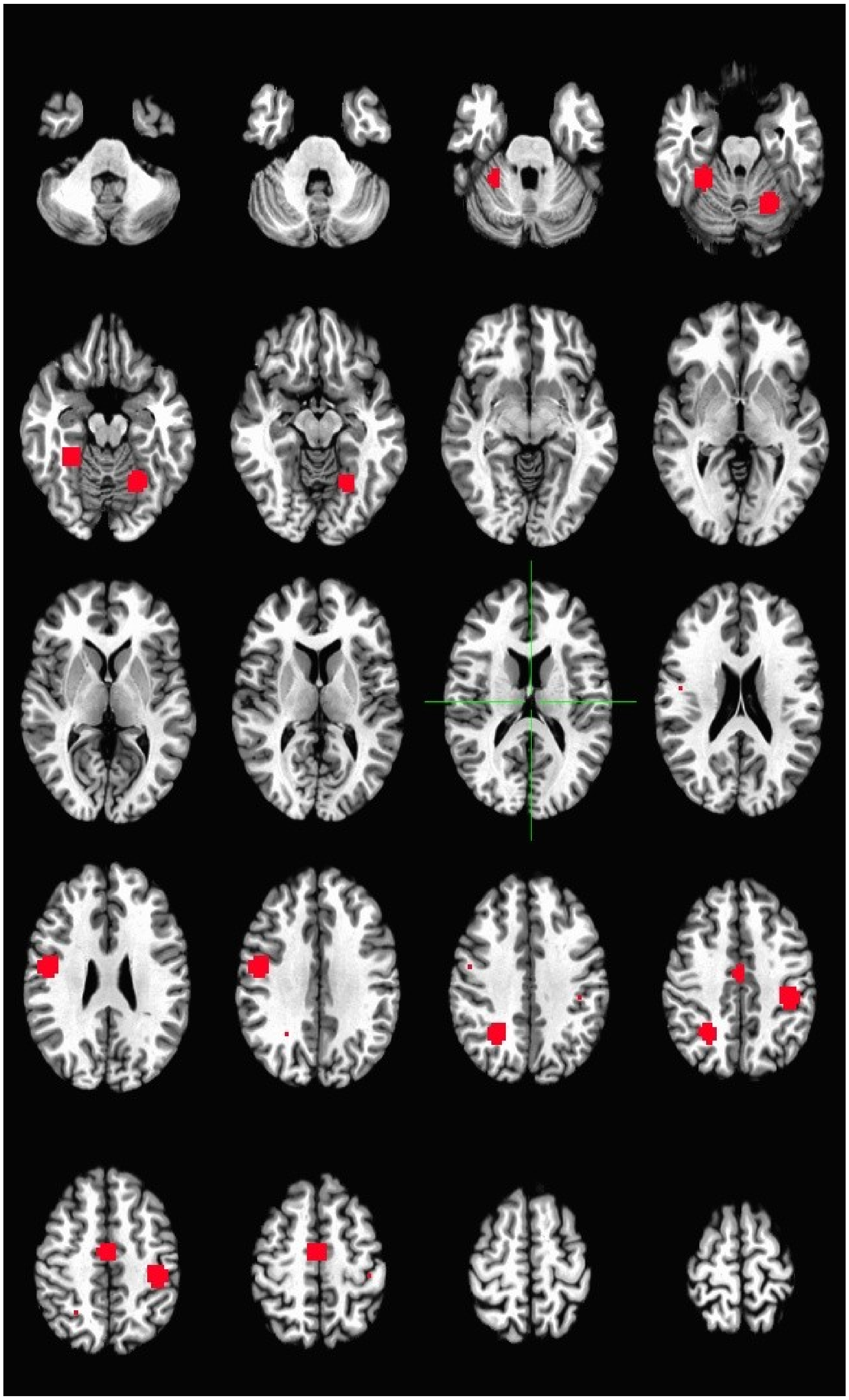

Supplementary Figure 5 The Paracentral lobule and the Somatosensory Network

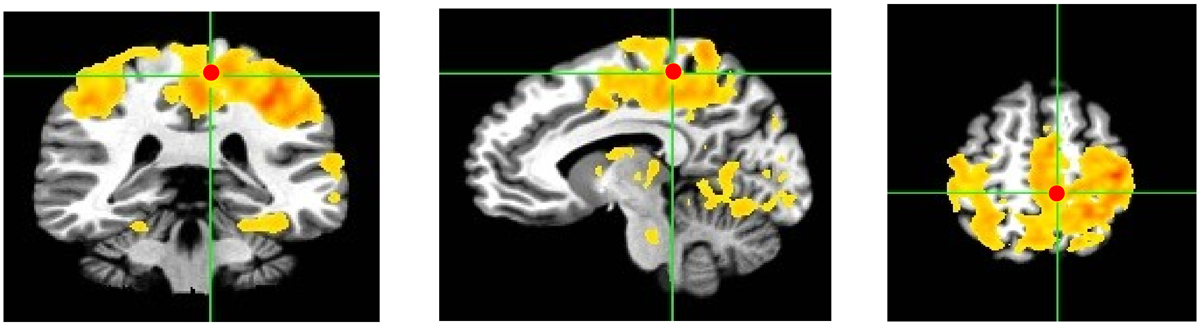

The region of the paracentral lobule characterized by higher gray matter density in bulimic patients compared to controls in Amianto et al. 2013 is indicated as a red circle; the yellow area is the connectivity of the somatosensory network (the procedure to obtain it is described in the paper).
